# Multicentric Retrospective Evaluation of Five Classic Infantile Pompe Disease Subjects Under Enzyme Replacement Therapy With Early Infratentorial Involvement

**DOI:** 10.3389/fneur.2020.569153

**Published:** 2020-11-25

**Authors:** Matteo Paoletti, Anna Pichiecchio, Giovanna Stefania Colafati, Giorgio Conte, Federica Deodato, Serena Gasperini, Francesca Menni, Francesca Furlan, Laura Rubert, Fabio Maria Triulzi, Claudia Cinnante

**Affiliations:** ^1^Advanced Imaging and Radiomics Center, Neuroradiology Department, Istituto di Ricovero e Cura a Carattere Scientifico Mondino Foundation, Pavia, Italy; ^2^Department of Brain and Behavioral Sciences, University of Pavia, Pavia, Italy; ^3^Oncological Neuroradiology Unit, Imaging Department, Istituto di Ricovero e Cura a Carattere Scientifico Ospedale Pediatrico Bambino Gesù, Rome, Italy; ^4^Fondazione Istituto di Ricovero e Cura a Carattere Scientifico Ca' Granda Ospedale Maggiore Policlinico, Neuroradiology Unit, Milan, Italy; ^5^Unit of Metabolic Disease, Istituto di Ricovero e Cura a Carattere Scientifico Ospedale Pediatrico Bambino Gesù, Rome, Italy; ^6^Pediatric Rare Diseases Unit, Department of Pediatrics, Fondazione Monza e Brianza per il Bambino e la sua Mamma, San Gerardo Hospital, Monza, Italy; ^7^Pediatric Highly Intensive Care Unit, Department of Pathophysiology and Transplantation, University of Milan, Istituto di Ricovero e Cura a Carattere Scientifico Ca' Granda Ospedale Maggiore Policlinico, Milan, Italy; ^8^Division of Inherited Metabolic Diseases, Department of Diagnostic Services, University Hospital of Padua, Padua, Italy; ^9^Department of Pathophysiology and Transplantation, University of Milan, Milan, Italy

**Keywords:** Pompe disease, IOPD, infantile-onset Pompe disease, brain MRI, ERT (enzyme replacement therapy), white matter (WM)

## Abstract

White matter (WM) abnormalities and ventricular enlargement in brain MRI are well-known features in infantile-onset Pompe disease (IOPD) in the era of enzyme replacement therapy (ERT). In this multicentric observational retrospective study, we report a small cohort of IOPD subjects under ERT treatment (*n* = 5, median age at MRI = 7.4 years, median period of treatment = 85 months) that showed the classic features of extensive supratentorial WM abnormalities but also unusual findings such as early infratentorial WM abnormalities and late supratentorial U-fiber involvement. Given the recent implementation of ERT and the rarity of the disease, a complete spectrum of presentation and understanding of progressive pathology in the brain of IOPD subjects in treatment remains underacknowledged. The availability of long-term follow-up of IOPD subjects under ERT treatment allows a better insight into the evolution of brain abnormalities in such disease.

## Introduction

Infantile-onset Pompe disease (IOPD) is a rare autosomal-recessive lysosomal glycogen storage disorder caused by a deficiency of the lysosomal enzyme acid alpha-glucosidase (GAA). The lack of lysosomal function of GAA results in accumulation of glycogen that deposits in body tissues, especially cardiac and skeletal muscles as well as the central nervous system (CNS). In the CNS, glycogen deposition has been documented not only in the neurons of cerebral cortex, brainstem, and anterior horns of the spinal cord, but also in oligodendrocytes and with relative sparing of cerebellar Purkinje cells (1, 2). The disease manifests in the first days or weeks of life and has a rapidly progressive course with early cardiac involvement, and in the natural history of the disease, death occurs within the age of 1 year.

Enzyme replacement therapy (ERT) was launched in 2006 and dramatically changed the natural disease course of cross-reactive immunological material (CRIM)-positive subjects, with CRIM-negative subjects showing only limited response to therapy or being more ventilator dependent (3, 4). Globally, ERT has markedly prolonged survival in IOPD but has also unveiled secondary aspects of the disease including white matter (WM) abnormalities in the CNS (3, 5–7). In the pre-ERT era, in fact, the very bad prognosis of IOPD impeded the possibility to detect brain abnormalities especially in the long term.

Ebbink et al. recently published the largest available IOPD cohort under ERT (*n* = 23), and, given the spectrum of brain abnormalities described, the disease course was divided “neuroradiologically” into three phases: a first phase with predominant periventricular WM involvement (starting at 2 years of age), subcortical WM and capsular involvement (from 8 years onwards), and later extension to the infratentorial WM (over the age of 11 years) (8). Nevertheless, the overall small number of brain studies in this field presumably still limits a comprehensive view of the entire neuro-pathological spectrum.

Our aim here is to cross-sectionally and longitudinally evaluate brain MRI findings in a small IOPD cohort on ERT treatment to better characterize brain abnormalities during therapy and disease evolution.

## Materials and Methods

Five subjects enrolled from different Italian centers (three patients from Milan, one from Monza, and one from Rome) were included in this multicentric retrospective observational study (range, 1 month to 16.5 years). The median age at MRI was 7.4 years; the median period of ERT treatment was 85 months (1 month to 16 years 6 months).

No ethical committee approval was necessary according to national regulations because this was a retrospective analysis of routinely collected anonymized clinical data. Informed consent for the use of clinical–radiological data acquired for routine clinical purposes in anonymized form was obtained from the children's parents.

All subjects had the classic form of Pompe disease and were CRIM positive (only in subject no. 1 was the CRIM positivity estimated based on genotype).

Eleven brain MRIs were performed: three subjects (2, 3, and 4) underwent one single scan, one subject (no. 1) had two scans, and another one (no. 5) underwent six serial scans. Conventional T1w and T2w sequences were acquired: Subject nos. 1, 4, and 5 also underwent MR spectroscopy (MRS) at intermediate TE (TE = 145 ms). MRIs were performed on either a 1.5-T or a 3-T scanner.

Brain MRIs were scored as follows: Evans index and maximum diameters of the III and IV ventricles were assessed and compared to age- and gender-matched references (6). The presence of WM signal abnormalities was assessed in the periventricular (PVWM) and subcortical WM, in the U-fibers, the *centrum semiovale*, corpus callosum (CC), anterior and posterior limb of the internal capsule (ALIC, PLIC), external capsule (EC), and the infratentorial WM (brainstem and/or cerebellar level). The gray matter nuclei (caudate, striatum, and thalamus) were also assessed.

Images were reviewed by two expert pediatric neuroradiologists (AP and CC) with more than 15 years of experience.

## Results

**Figure 1:**
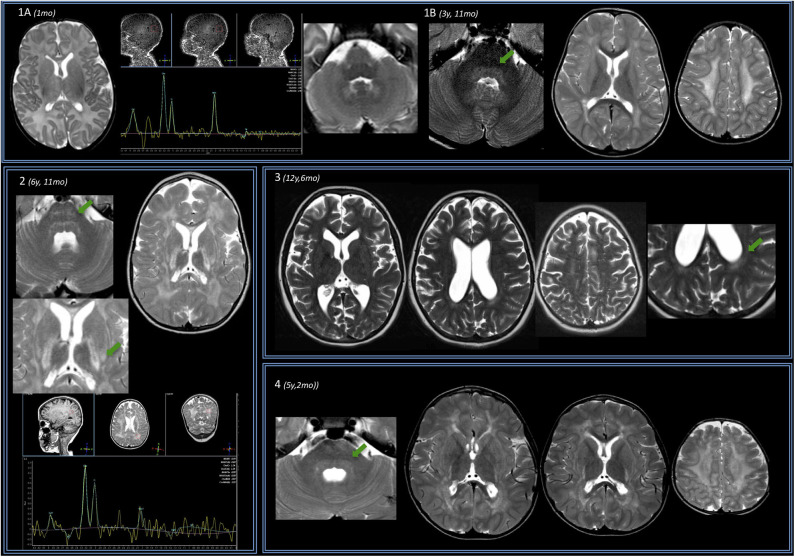
Brain MRIs of the IOPD cohort. Four subjects are displayed (same order as reported in Table 1). **(A)** Subject no. 1. Perinatal structural brain MRI scan is unremarkable; MRS already shows NAA depletion. **(B)** At later follow-up around the age of 4, axial T2w images show mild hyperintensity of the dentate nuclei hilum and of the dorsal pons (arrow). At the supratentorial level, periventricular WM of both the *centrum semiovale* T2 hyperintensities are evident. (2) Subject no. 2. Early after the age of 6 years (6 years 11 months), a mild T2w hyperintensity is evident along the transverse pontine fibers (arrow) and also in the dorsal pons bilaterally. In the supratentorial structures, there is a diffuse T2 hyperintensity of the periventricular and subcortical WM with sparing of the U fibers. The posterior limb of the *internal capsulae* has a marked T2 signal increase. A T2 hyperintensity is also evident at the level of the tail of putamen, bilaterally (arrow). Spectroscopy performed at the level of periventricular posterior WM shows reduction of the NAA. (3) Subject no. 3 at the age of 12 years with mild WM involvement: T2w images show only a mild involvement of the posterior periventricular WM (arrow) and of *centrum semiovale* bilaterally. (4) Subject no. 4. Before the age of 6, a mild T2-signal hyperintensity is evident at the level of dorsal pons (arrow) and along both cortical spinal tracts. Supratentorially, MRI showed a T2-signal hyperintensity of the fronto-parietal WM (with sparing of U fibers) and along the posterior limb of the internal capsule and the external capsule bilaterally is evident. There is evidence of initial brain softening at the level of both *centrum semiovale*.

**Figure 2:**
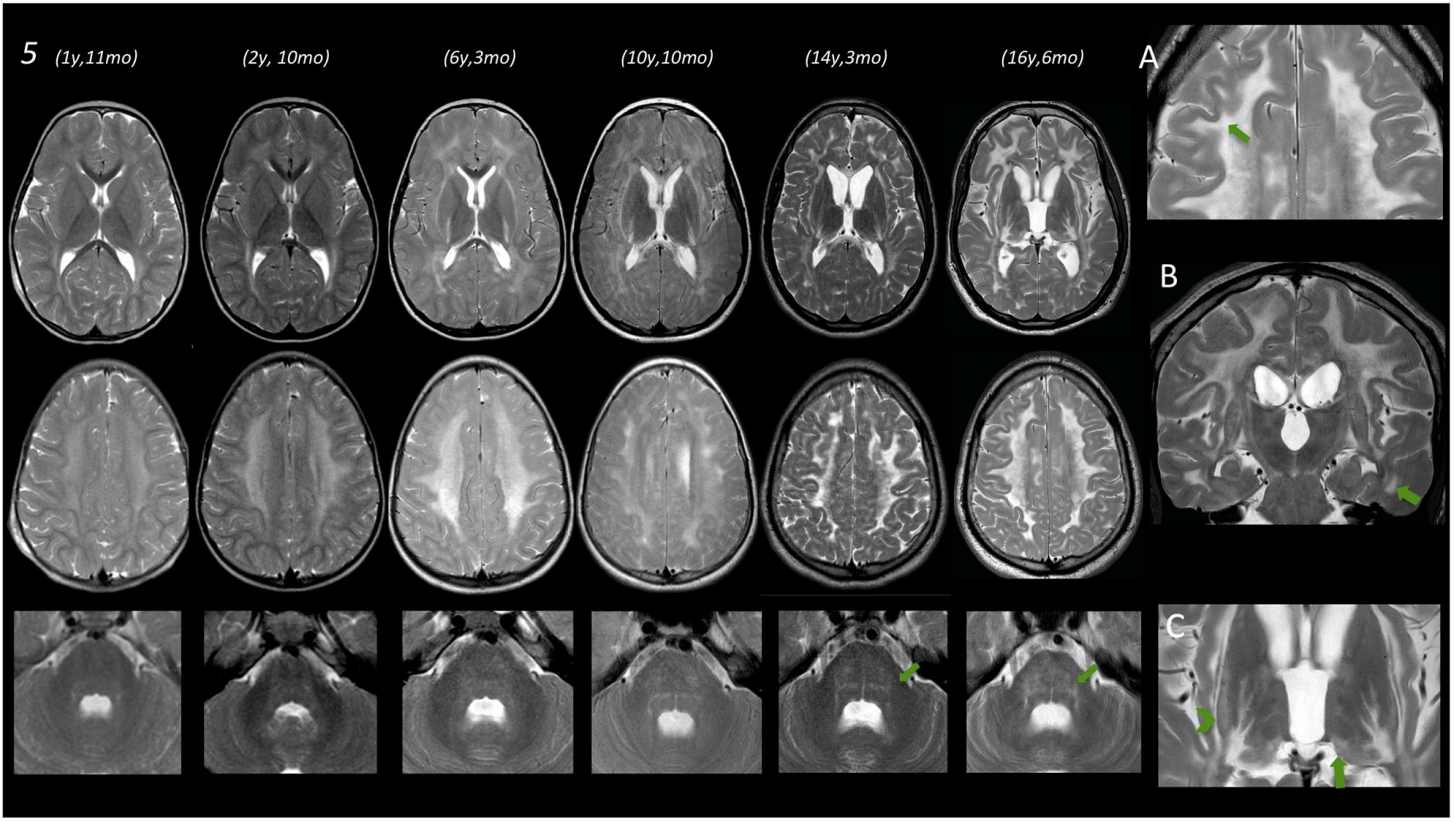
Brain MRIs of subject no. 5. In the left part of the figure, MRIs of three different levels (deep nuclei, centrum semiovale, and middle cerebellar peduncles) are displayed. Under the age of 3 years, only a very mild T2 hyperintensity is evident at the level of the periventricular WM. Subsequent MRI scans show a progressive marked diffuse T2 hyperintensity of the WM also involving the posterior limb of the internal capsule (PLIC) as well as the external and the extreme capsule bilaterally. Infratentorially, only a mild T2 hyperintensity is evident at the age of 6 years 3 months at the level of the dorsal pons that becomes more pronounced at the age of 10 and 14–16 years. **(A)** Magnification of axial T2 image acquired after the age of 16, showing a remarkable involvement of the U fibers (arrow). (**B)** Coronal T2 image acquired at the age of 16 years showing involvement of temporal WM (arrow) and also T2 hyperintensity of corticospinal tracts. **(C)** Magnification of axial T2 images at the age of 16 showing T2 hyperintensities at the level of the tail of the putamen (arrowhead) and the level of the pulvinar (arrow).

The brain MRI findings are reported in Table 1. Exemplificative brain MRI images are reported in Figures 1, 2.

**Table 1:** Radiological of the five IOPD subjects under enzyme replacement therapy (ERT).

**#**	**MRI time point**	**ERT start (dose) (mg/kg/week)**	**Age at MRI (years, months)**	**ERT total time at MRI date (years, months)**	**Evans index**	**III ventr. (mm)**	**IV ventr. (mm)**	**PVWM**	**Centrum semiovale**	**SWM**	**WM pseudocysts**	**Gradient**	**CC**	**U-f**	**ALIC**	**PLIC**	**EC**	**Infratentorial WM**	**GM**
1	t1	7 days (20 mg/kg/week)	1 month, 6 days	1 month	0.28 (=)	5 (+)	5.7 (=)	0	0	0	0	No	0	0	0	0	0	0	0
	t2	=	3 years, 11 months	4 years	0.25 (–)	7 (+)	9.8 (=)	1	1	1	1	No	0	0	0	0	0	1	0
2	t1	4 months (20 mg/kg/2 weeks)	6 years, 11 months	6 years, 7 months	0.27 (=)	6.2 (+)	12.6 (+)	1	1	1	1	No	1	0	1	1	1	1	Putamen R/L Thalamus R/L
3	t1	8 months (20 mg/kg/week)	12 years, 6 months	11 years, 10 months	0.33 (+)	8 (+)	14 (+)	1	1	0	0	Parietal > frontal	0	0	0	0	0	0	0
4	t1	3 months (40 mg/kg/week)	5 years, 2 months	4 years, 11 months	0.25 (=)	7 (+)	12.4 (+)	1	1	1	1	No	1	0	1	1	1	1	Putamen R/L
5	t1	4 months (40 mg/kg/2 weeks)	1 year, 11 months	1 year, 7 months	0.25 (–)	5.3 (=)	9.3 (–)	1	1	1	0	Fronto-parietal > temporal	0	0	0	0	0	0	0
	t2	=	2 years,10 months	2 years, 6 months	0.25 (–)	5.4 (–)	9.4 (–)	1	1	1	0	=	0	0	0	0	0	0	0
	t3	=	6 years, 3 months	5 years, 11 months	0.23 (–)	5.7 (–)	10.6 (=)	1	1	1	1	=	1	0	0	1	1	1 (mild)	0
	t4	=	10 years, 10 months	10 years, 6 months	0.28 (+)	11.9 (+)	10.9 (=)	1	1	1	1	=	1	1	0	1	1	1	Thalamus R/L
	t5	=	14 years, 3 months	13 years, 11 months	0.30 (+)	12.2 (+)	13.2 (+)	1	1	1	1	=	1	1	1	1	1	1	Thalamus R/L Putamen R/L
	t6	=	16 years, 6 months	16 years, 2 months	0.31 (+)	12.4 (+)	13.3 (+)	1	1	1	1	Also temporal WM	1	1	1	1	1	1	=

*For each subject and time point, details are reported. All subjects except subject no. 2 are alive at the moment of paper writing. 0, absent; 1, present; MRI, Magnetic Resonance Imaging; PVWM, periventricular white matter; SWM, subcortical white matter; WM, white matter; CC, corpus callosum; ALIC, internal capsule anterior limb; PLIC, internal capsule posterior limb; EC, external capsule; GM, gray matter. WM and GM abnormalities are evident as T2 hyperintensities (see also Figures 1, 2). Regarding Evans Index and the measures of III and IV ventricle, absolute numbers are reported (the ratio for the Evans index, mm for III, and IV ventricle); additionally “+,” “=,” or “–” are reported for comparison to the references as given by Sari et al*.

### Ventricular Size

The Evans index was normal or even reduced vs. controls in three cases. It increased in one subject (no. 3), and it progressively increased to pathologic enlargement in subject no. 5. The diameters of III and IV ventricles were generally increased quite early and/or were increased at later ages (especially subject no. 5).

### WM Abnormalities

The only perinatal structural MRI obtained (no. 1, Figure 1A) was unremarkable. The two MRIs acquired at 2–4 years of age already showed diffuse signal alterations of the fronto-parietal PVWM, SWM, and *centrum semiovale* sparing the U-fibers. MRI acquired at subsequent ages (no. 5, also after the age of 12 years) showed marked progression of WM abnormalities with a “pseudocystic pattern” at the periventricular level bilaterally as well as involvement of the internal and external capsulae and ultimately the U-fibers (Figures 1A,B).

WM abnormalities were globally symmetric in all subjects, with no predominant side. A gradient with fronto-parietal predominant involvement and relative sparing of occipital and temporal lobes was evident. In subject no. 5 at the age of 16.5 years (last follow-up available), temporal WM involvement was also shown.

No WM abnormality was found at the infratentorial level in subjects below the age of 3 years. Pontine involvement (especially the arcuate and transverse fibers) as well as WM adjacent to IV ventricle and the dentate nuclei hilum showed T2w hyperintensities at the age of ~4 years (subject no. 1) and in two cases around the age of 5–6 years (no. 2 and 4). Infratentorial abnormalities were symmetric in all subjects and progressed over time in the long-term follow-up of subject no. 5 (Figure 2).

### Deep Nuclei

Putaminal abnormalities in the shape of T2 hyperintensity at the level of the tail were evident at the age of 5, 6, and 10 years in three cases (2, 3, and 5); thalamic involvement (especially pulvinar, again seen as T2 hyperintensity), with progressive reduction of global thalamic volume, was only shown in subject no. 5 starting at the age of 10 (Figure 2).

### MR Spectroscopy

Spectroscopy showed a mild decrease of N-acetyl-aspartate (NAA) in PVWM in three cases in which it was performed (Figure 1). It was detected at perinatal time in subject no. 1 and was also evident at 5 years (subject no. 2). Such finding was confirmed at the age of 14 when MRS was first performed in subject no. 5, with a subtle further reduction of NAA at the last follow-up (age of 16.5 years) (not shown).

### Evolution Over Time

We only have longitudinal data for subject nos. 1 and 5. In subject no. 1, no abnormality was found apart from mild decrease of NAA in the perinatal MRI; at the age of almost 4 years, both infra- and supratentorial WM abnormalities were evident.

In subject no. 5, given the number of serial MRIs available, we observed a progression of WM and GM abnormalities, both at supra- and infratentorial level over time.

### Clinical Findings

Due to the retrospective multicentric collection of cases and to the lack of a common clinical evaluation protocol, clinical data for our cohort are quite heterogeneous.

The cognitive evaluation for subject no. 1 at the time of her clinical follow-up (around 7 years, no MRI performed at this time point) was normal with an intelligence quotient (IQ) of 119 (Wechsler Intelligence Scale for Children version IV, WISC-IV).

Subject no. 2 had such severe motor impairment [severe hypotonia, ventilator-dependent; level 5 of the Gross Motor Function Classification System (GMGCS) scale] that a standard cognitive evaluation could not be collected with a standardized test. The last available Vineland Adaptive Behavior Scale IQ score was <55. The subject ultimately died at the age of 10 (ventilator assisted, parenteral nutrition, no external interaction expect for eye movements).

Subject no. 3 showed a persistent selective mutism toward healthcare providers. To assess cognitive development, non-verbal psychometric tests have been used (Raven Matrices and WISC-IV non-verbal items only), respectively, at 11 and 12 years of age (the last time point close to the MRI). In both cases, IQ was within the normal range.

Subject no. 4 (child with tracheostomy) scored 93 in the Leiter-R score.

Subject no. 5 at 10 years of age reported an IQ of 56 (WISC-III) and, later, at 14 years of age, reported a worsening in cognitive function with an IQ of 44 (WISC-III) and 56 at the Leiter-R scale. Subject no. 5 also had multiple subependymal heterotopic nodules with history of seizures, in therapy with levetiracetam (presumably as an occasional finding). EEG repeatedly reported diffuse slowing with sharp waves in the anterior derivations, which was interpreted as consistent with diffuse leukoencephalopathy. No genetic test was performed.

All available clinical data are summarized in Table 2.

**Table 2:** Clinical data of the IOPD cohort evaluated at each time point within 1 month from each brain MRI.

**Subject**	**Time point**	**Age at MRI/clinical evaluation**	**Duration of ERT therapy (years, months)**	**Ventilatory assistance**	**Ability to walk**	**Verbal status**	**Cognitive evaluation**
#1	t1	0–3 months	1 month	No	N/A	N/A	N/A
//	t2	<6 years	4 years	No	Yes	Normal with rhinolalia	Griffiths' IQ 93
//	t3	>6 years	7 years	No	Yes with stepping gait	Normal with rhinolalia	WISC-IV IQ119
#2	t1	>6 years	6 years 7 months	yes	GMFCS level V	Only facial mimics and finger movements	Not assessable; only VABS scale <55
#3	t1	>12 years	11 years, 10 months	Yes, NIV from the age of 4 years	Autonomous from 15 months of age, then lost at 4 years	Selective mutism with sanitary operators	RPM: IQ normal WIC IV (non-verbal subtests) normal: PRI 102; PSI 115; VABS: total score 88
#4	t1	<5 years	4 years, 11 months	Yes (24 h/day by tracheostomy)	No (tetraparesis)	Only through facial mimics and few words	Leiter-R Scale IQ = 93
#5	t1	<3 years	1 year, 7 months	No	Yes	Normal with rhinolalia	N/A
//	t2	<3 years	2 years, 6 months	No	Yes	Normal with rhinolalia	N/A
//	t3	> 6 years	5 years, 11 months	No	Yes	Normal with rhinolalia	N/A
//	t4	> 6 years	10 years, 6 months	No	Yes	Normal with rhinolalia and mild dysarthria	RPM 3° percentile WISC III = IQ 56
//	t5	> 12 years	13 years, 11 months	No	Yes	Rhinolalia and mild dysarthria	RPM 9° percentile WISC III = IQ 50 Leiter = IQ 67
//	t6	>16 years	16 years, 6 months	No	No	Few faint words, impaired pronunciation and severe dysarthria	WISC III IQ 44 Leiter IQ 56 Scale severe psychomotor slowdown, ideomotor slowdown, loss of any autonomy

*WISC, Wechsler Intelligence Scale for Children; RPM, Raven Progressive Matrices; GMFCS, Gross Motor Function Classification System; N/A, not assessed; VABS, Vineland Adaptive Behavior Scales; IQ, Intelligence quotient; NIV, non-invasive ventilation*.

## Discussion

In the present study, we wanted to explore the neuroradiological picture of a small Italian cohort of IOPD subjects under ERT treatment. The known extensive supratentorial WM involvement described by Ebbink et al. in their cohort (*n* = 23 cases) (8) was globally confirmed in our five subjects along with capsular and corticospinal tract involvement. Ventricular enlargement was also confirmed (with additional comparison to the references for age and sex) (9).

In contrast to what was already reported in the literature, we now reported novel findings regarding the early infratentorial WM involvement in the first years of life (under the age of 6) and also late supratentorial U-fiber involvement in the long-term follow-up of one subject.

Infratentorial WM abnormalities have previously only been described in advanced disease stages usually after the age of 11 (8). In our cohort, however, three subjects presented with pontine involvement already at 4 years of age (subjects nos. 1, 4, and 5). The occurrence of late infratentorial WM abnormalities may be interpreted, in a very conservative way, as a consequence of progressive supratentorial loss of substance. Conversely, our findings in the early years of life suggest that infratentorial abnormalities can only be partially interpreted as secondary to supratentorial WM loss, as they can be present quite early and also progress over time in parallel to supratentorial abnormalities.

Secondly, our long-term follow-up of a single subject ultimately demonstrated that supratentorial U-fibers might be effectively involved in the disease, suggesting that no specific sparing of WM would occur over long-term progression. Also, this finding can be considered as novel, as in other previous studies, U-fibers sparing was commonly reported (7, 8).

The finding of NAA depletion at MRS seems to suggest that neuronal loss is evident from birth onwards, and it is still evident at long-term follow-up along with progressive extension of WM abnormalities.

Extensive progressive WM involvement and neuronal loss seen in subject no. 5 mirrored a slow progressive cognitive decline. This seems to indicate that brain alterations may not be an epiphenomenon of a delayed myelination but rather be the direct result of the glycogen deposition (2, 6, 7, 10–12), even during ERT treatment. ERT demonstrated significant improvement in survival, cardiac, and motor outcome of IOPD subjects in therapy (11), but it cannot pass the blood–brain barrier. Glycogen has been shown to deposit vastly in the CNS, with special mention to glial cells (1, 2), and it is known that glycogen deposition recalls water. We may hypothesize that WM abnormalities could at least partially be interpreted as due to this process and not to a true delayed myelination. Our feeling is that, with more brain MRI follow-ups available in the future, the CNS abnormalities are becoming more and more recognized in the IOPD population.

Clinical evaluation of IOPD subjects, as already reported in the methods, was quite heterogeneous in our multicentric and retrospective study, lacking a common prospective protocol. As a matter of fact, the clinical (and above all cognitive) evaluation of this cohort was beyond the scope of the study (and thus it is not an endpoint of the study) in which we wanted to explore the aspect and the evolution of brain abnormalities in IOPD during ERT therapy. Despite such heterogeneity of data and a difficult comparability of the cognitive profile of IOPD subjects, the current dataset confirmed that cognitive impairment is evident in IOPD subjects undergoing ERT, despite therapy (12). We also found that, in the only subject with a long follow-up, the cognitive profile declined over time. As already suggested by Ebbink et al. the occurrence and progression of WM in IOPD subjects have presumably an impact on intelligence and neuropsychological functions, with the extensive involvement of fronto-parietal areas that are connected to such superior function (8).

Limitations apply to our study: first of all, the limited number of cases; secondly, the lack of prospective data and of a standard clinical protocol of evaluation; thirdly, the availability of follow-up only in two cases (of which only one is a long-term follow-up).

In conclusion, our observational retrospective multicentric data in a small cohort of IOPD suggest how brain manifestations of the disease during ERT therapy are still quite underacknowledged. Larger case series also with clinical correlation and preferably prospective end standardized follow-up protocols of study are still needed to achieve full insight into the disease CNS manifestations.

## Data Availability Statement

The raw data supporting the conclusions of this article will be made available by the authors, without undue reservation.

## Ethics Statement

Ethical review and approval was not required for the study on human participants in accordance with the local legislation and institutional requirements. Written informed consent to participate in this study was provided by the participants' legal guardian/next of kin.

## Author Contributions

AP, CC, and MP contributed to the study conception and design, material preparation, data collection, and presentation were performed by MP. The first draft of the manuscript was written by MP, AP, and CC. All authors commented on previous version of the manuscript. All authors read and approved the final manuscript.

## Conflict of Interest

The authors declare that the research was conducted in the absence of any commercial or financial relationships that could be construed as a potential conflict of interest.

## Abbreviations

ERT: enzyme replacement therapy
IOPD: infantile-onset Pompe disease
MRI: magnetic resonance imaging
WM: white matter
PVWM: periventricular WM
SWM: subcortical WM
CC: corpus callosum
ALIC: internal capsule anterior limb
PLIC: internal capsule posterior limb
EC: external capsule
GM: gray matter.
